# Tetrameric Transthyretin as a Protective Factor Against Alzheimer’s Disease

**DOI:** 10.1007/s12035-024-04442-8

**Published:** 2024-08-27

**Authors:** Camilla Corino, Alberto Aimo, Marco Luigetti, Lidia Ciccone, Yu Fu Ferrari Chen, Giorgia Panichella, Veronica Musetti, Vincenzo Castiglione, Giuseppe Vergaro, Michele Emdin, Maria Franzini

**Affiliations:** 1 Health Sciences Interdisciplinary Center, Scuola Superiore Sant’Anna, Piazza Martiri Della Libertà 33, 56127 Pisa, Italy; 2 Cardiology Division, Fondazione Toscana Gabriele Monasterio, Pisa, Italy; 3 Fondazione Policlinico Agostino Gemelli IRCCS, UOC Neurologia, Università Cattolica del Sacro Cuore, Rome, Italy; 4 Department of Pharmacy, University of Pisa, Pisa, Italy; 5 Careggi University Hospital, Florence, Italy; 6 Department of Translational Research On New Technologies in Medicine and Surgery, University of Pisa, Pisa, Italy

**Keywords:** Transthyretin, Alzheimer’s disease, Neuroprotection, Amyloidosis, Therapeutic strategies

## Abstract

Transthyretin (TTR) is a tetrameric protein traditionally recognized for its role in transporting thyroxine and retinol. Recent research has highlighted the potential neuroprotective functions of TTR in the setting of Alzheimer’s disease (AD), which is the most common form of dementia and is caused by the deposition of amyloid beta (Aβ) and the resulting cytotoxic effects. This paper explores the mechanisms of TTR protective action, including its interaction with Aβ to prevent fibril formation and promote Aβ clearance from the brain. It also synthesizes experimental evidence suggesting that enhanced TTR stability may mitigate neurodegeneration and cognitive decline in AD. Potential therapeutic strategies such as small molecule stabilizers of TTR are discussed, highlighting their role in enhancing TTR binding to Aβ and facilitating its clearance. By consolidating current knowledge and proposing directions for future research, this review aims to underscore the significance of TTR as a neuroprotective factor in AD and the potential implications for future research.

Transthyretin (TTR) is a tetrameric protein secreted in the plasma and cerebrospinal fluid (CSF) acting as a carrier of thyroxine and retinol binding protein (RBP). Besides its role in the pathogenesis of TTR amyloidosis (ATTR), a growing body of evidence points to a role of TTR in the development of another form of amyloidosis, namely Alzheimer’s disease (AD).

The prevalence of dementia has increased rapidly in recent years. In 2020, over 55 million people were affected by the syndrome, and this number is expected to double within 20 years, primarily due to increased life expectancy, though other risk factors also play a crucial role [2]. Alzheimer’s disease (AD) is the most common form of dementia, accounting for approximately 70% of cases [1]. The diagnosis of AD in vivo is based on criteria established in 1984 [4] and revised in 2011 [5]. Since neuropathological changes develop years before symptoms appear [6, 7], a diagnosis based on clinical criteria cannot capture the prodromal phase, making it difficult to prevent or slow disease progression. Additionally, AD symptoms are quite similar to those of other forms of dementia [8], and a reliable diagnostic method remains an unmet need. Some progress has been made, particularly with the introduction of biomarkers for cerebrospinal fluid (CSF) analysis [9] and Aβ PET molecular imaging [10].

Amyloid fibrils are formed because of incorrect folding of protein rich in β-sheets, and the nature of the aggregating peptide determines the features of the specific disease. Kinetically unstable conformations of TTR have been linked to the pathogenesis of ATTR [11], while AD is believed to develop when amyloid beta protein (Aβ) deposits in the extracellular matrix following proteolytic processing of a transmembrane protein, amyloid precursor protein (APP) [12].

Interestingly, TTR is a carrier and chaperone of cytotoxic Aβ peptides and therefore has anti-amyloidogenic and neuroprotective activities in the central nervous system (CNS) [13]. In AD, the stability and functionality of TTR seems compromised, possibly leading to an accumulation of Aβ fibrils and, subsequently, to neurodegeneration and cognitive decline. TTR stabilization would then have beneficial effects also on AD progression [14]. In this review, we provide an overview of the experimental evidence supporting this hypothesis, and we suggest some possible perspectives for future research.

## Transthyretin: Molecular Structure and Stability

**Fig. 1:**
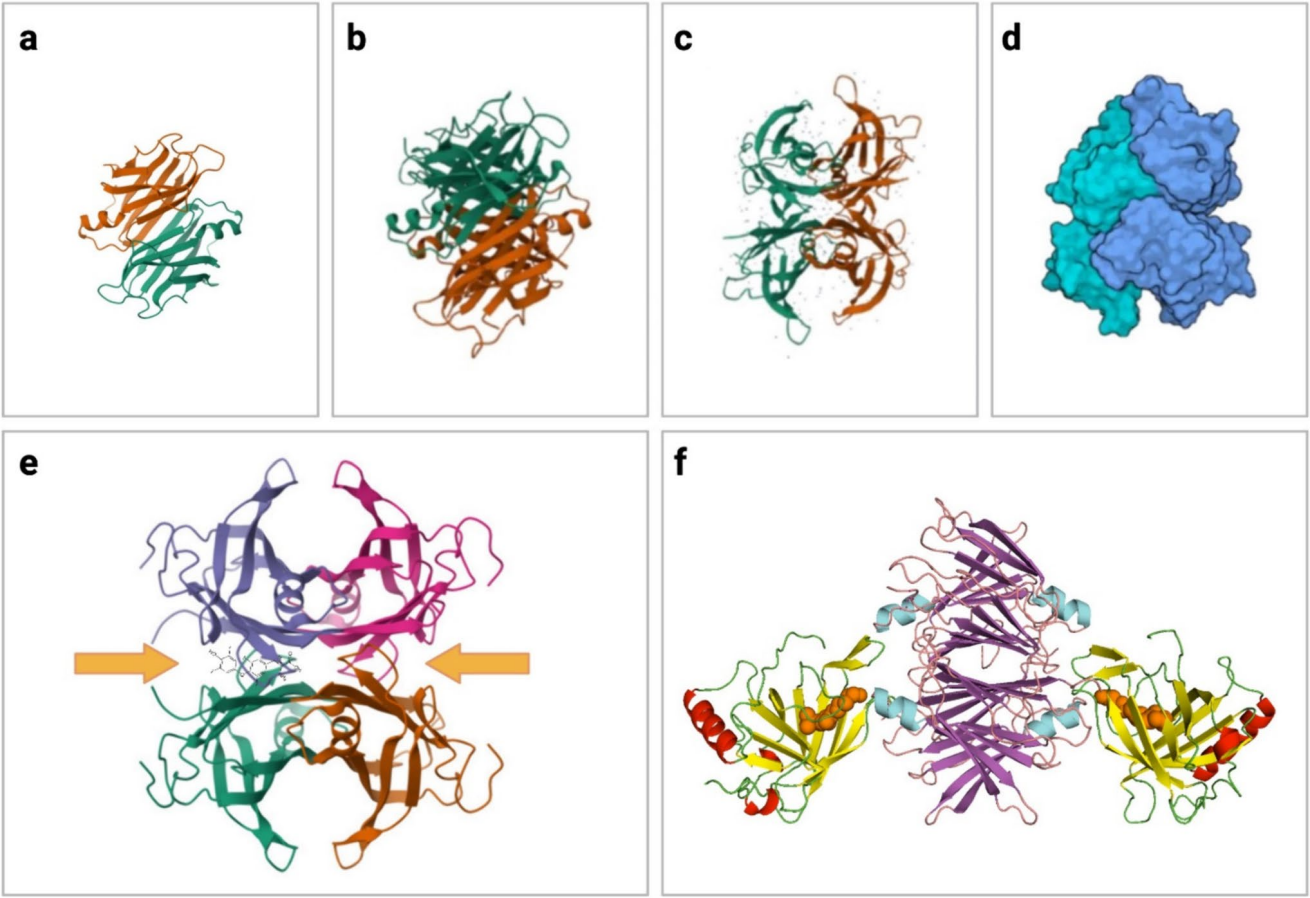
Tridimensional structure of transthyretin (TTR). **a** Cartoon diagram of TTR dimer; **b–c** TTR tetramers seen in different projections; **d** contact surfaces of the 4 TTR monomers; **e** TTR complexed with a thyroxine (T4) molecule bound in the inner hydrophobic channel (yellow arrows); **f** cartoon diagram of TTR complexed with two retinol binding protein (RBP) molecules (yellow/red/green structures), carrying one retinol each (orange structures). Tridimensional protein structures taken from RCSB PDB database (protein IDs: 2PAB, 3W3B, 1ICT, 1QAB), access date 2 April 2024

TTR is a small protein (55 kDa) composed of 4 identical monomers [15] encoded by a gene on chromosome 18 [16]. It has a globular structure with two central hydrophobic channels, where two molecules of T4 can bind (Fig. 1). In physiological conditions, only one T4 is bound, because of the negative cooperativity between the two sites [17]. T4 binding stabilizes the TTR tetramer [18]. RBP binds in another site to the external surface of TTR. Each dimer has four possible binding sites for RBP, but only two molecules can bind because of steric hindrance [19] **(**Fig. 1). Retinol binding induces conformational changes in RBP that increase its affinity for TTR. Binding of either T4 or RBP inhibits TTR destabilization and amyloid formation [20].

Decreases in pH, ageing, metal cations (particularly calcium ions), and oxidation may reduce TTR stability [21], but the main causes of TTR destabilization, which leads to the reaggregation of monomers in cytotoxic quaternary structures, and, ultimately, to ATTR amyloidosis, are single point mutations in the *TTR* gene [22]. More than 140 mutations with autosomal dominant transmission have been reported, and just few of them do not cause TTR dissociation and fibril formation. Only three mutations are known to increase TTR stability [23].

## Synthesis and Catabolism

**Fig. 2:**
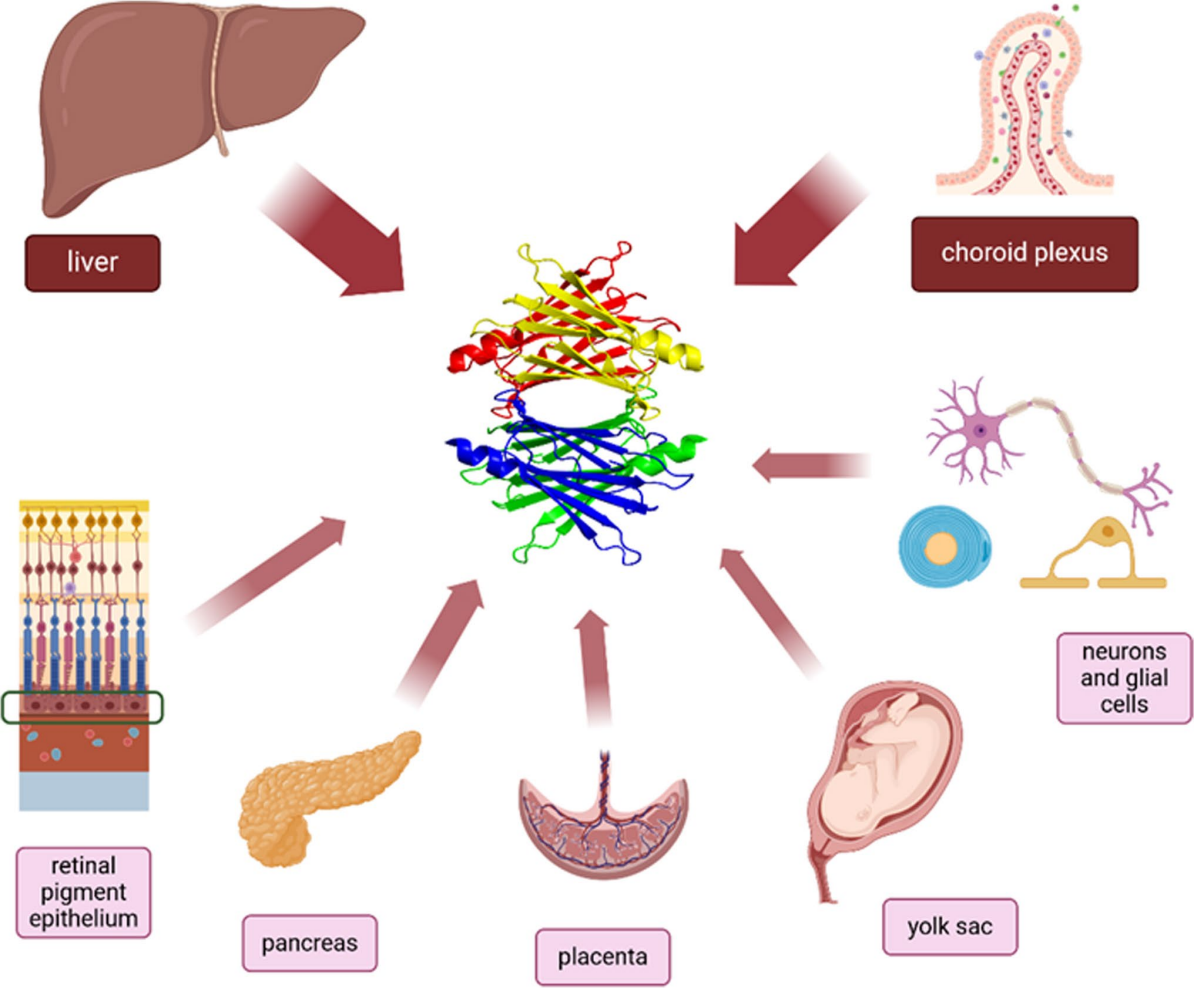
Sites of transthyretin (TTR) production

TTR is a highly conserved protein mainly secreted by the liver in the blood flow and the choroid plexus (CP) in the cerebrospinal fluid (CSF) [24]. The liver secretes up to 90% of TTR in humans [23]. *TTR* gene expression in the liver is modulated by hepatocyte nuclear factors (HNF) and is reduced by inflammation or malnutrition [25]. TTR concentration in the CSF is lower than in the blood, but TTR represents about 25% of the total protein content of the CSF and is synthesized much faster [26]. *TTR* expression in the choroid plexus is not modulated by HNF and is not affected by systemic inflammation [23]. TTR is also synthesized in small amounts in other tissues (Fig. 2).

TTR produced by the placenta [27] and yolk sac [28] is crucial to transport maternal T4, which is required for embryonal development. In the pancreas, TTR is mainly synthesized by α-cells [29], and promotes glucose-induced insulin release [30]. In the retina, TTR is produced by the retinal pigment epithelium together with RBP [31, 32] and transports retinol to photoreceptors [23]. Low TTR levels may be synthesized also by the skeletal muscle (where TTR promotes myoblast differentiation and muscle growth) [33, 34] and in other sites (heart, spleen, stomach), where its functions are still unknown [24].

Importantly, TTR is also secreted in the peripheral nervous system (PNS) by the Schwann cells [35] and in the CNS by neurons and oligodendrocytes [36], especially in the hippocampus [37], where it exerts neuroprotective functions and lowers the production of amyloid aggregates [38], characteristic of AD.

## Physiological Roles of TTR: Thyroxine and Retinol Carrier and Proteolytic Activity

TTR carries only 15% of protein-bound T4 in the plasma, the rest being transported by thyroxin-binding globulin and albumin, while TTR carries 80% of T4 in the CSF [39]. Plasma RBP is secreted by hepatocytes and represents the unique specific transporter for retinol in the bloodstream, 95% circulating in complex with TTR, to avoid its renal filtration [40]. The relevance of plasma TTR for normal development and organ functioning is debated, also because selective deletion of plasma TTR cannot be achieved. Furthermore, studies on TTR knock-out (KO) mice showed either normal organ development [41] or delayed bone growth, delayed development of intestine, and altered CNS development, which were attributed primarily to a deficiency of thyroid hormones within tissues [42].

TTR may also cleave the C-terminal of apolipoprotein A-I (ApoA-I). This cleavage reduces cholesterol efflux by ApoA-I and increases its amyloidogenic potential [43]. Additionally, two possible substrates of TTR have been identified in the central nervous system: neuropeptide Y [44], a molecule with anti-inflammatory and neuroprotective functions [45], and amyloid β peptide (Aβ), which plays a key role in AD pathogenesis. TTR is one of the main Aβ-binding proteins and is able to cleave both its soluble and aggregated forms, decreasing its toxicity [46].

## TTR as a Neuroprotective Factor

TTR was first found in the PNS, and specifically in the endoneurial fluid, either coming from the CSF after crossing the blood-nerve barrier, or synthesized by the glial cells of the dorsal root ganglia (DRG) and by the Schwann cells [35]. Neuroprotective effects of TTR in the PNS were postulated based on observations on TTR KO mice, which showed sensorimotor impairment and decreased ability to regenerate sciatic nerve after crush [48]. TTR may promote nerve regeneration and neurite outgrowth following internalization in DRG neurons through the receptor megalin and activation of an intracellular pathway or by stimulating axonal transport (which is impaired in TTR KO mice) [49]. The neuritogenic TTR activity seems to be independent of its carrier function, since a TTR variant with decreased transport ability maintain its neuritogenic role [49], and not essential for neuronal survival, as the lack of TTR does not associate with increased neuronal loss [48].

In the CNS, TTR is mostly secreted by the epithelial cells of the choroid plexus, despite it can also be expressed by neurons and oligodendrocytes [36]. It exerts neuritogenic activity in hippocampal neurons, as well as neuroprotection in case of cerebral ischemia [50] or AD [38]. In vitro evidence demonstrated that TTR reduces the formation of harmful Aβ aggregates by proteolysis in cultured hippocampal neurons, protecting them from neurotoxicity [38]. Studies on *TTR* KO mice showed the lack of TTR leads to an accelerated memory deficit with age [51], and, conversely, TTR expression is decreased in rats with age-related memory impairment [52]. These findings confirm the hypothesis that TTR play a key role in disorders characterized by memory loss, such as AD and other dementia, possibly also through mechanisms other than its binding to Aβ [53]. For example, TTR KO mice show also an impaired neuronal differentiation in the subventricular region with a shift from neurodifferentiation towards oligodendrogenesis, which results in a hypermyelination of the brain [54]. Proliferation, survival and differentiation of oligodendrocytes is mediated by the phosphatidylinositol 3-kinase (PI3K)/Akt and extracellular signal-regulated protein kinases 1 and 2 (ERK1/2) pathways [55]. TTR KO mice exhibit increased Akt phosphorylation in oligodendroglial lineage cells, suggesting a possible mechanism of action of TTR [53]. Conversely, TTR binds and activates the insulin-like growth factor-1 receptor/Akt signaling pathway in hippocampal neurons [56], pointing to distinct roles of TTR in different cells and brain regions.

## Central Nervous System Involvement in ATTR Amyloidosis and Alzheimer’s Disease

Amyloidosis comprises several pathologies characterized by the accumulation of cytotoxic, insoluble fibrils in different tissues. Growing evidence in recent years has pointed out an involvement of TTR in the development of two amyloidotic disorders—transthyretin amyloidosis (ATTR) and AD—along with its neuroprotective functions. Amyloid deposition in ATTR amyloidosis occurs first in leptomeningeal vessels, arachnoid and pia, followed by perforating cortical vessels and the subpial region. Afterwards, subependymal deposition and involvement in basal ganglia vessels close to the ependymal lining develops. The two structures affected earlier by cerebral amyloid angiopathy (CAA) are the brainstem and the spinal cord [57]. Symptoms of CNS involvement develop at least 14 years after the onset of symptomatic systemic disease, as a frequent complication especially in patients with hereditary ATTR amyloidosis and the Val30Met mutation [58]. Its manifestations include transient focal neurological episodes and, less commonly, intracerebral hemorrhages, ischemic stroke, and cognitive deterioration [59–61]. The Hisayama study reported ATTR amyloidosis in 23% and CAA in 36% of autopsies of elderly adults. The prevalence of both ATTR amyloidosis and CAA increased in patients with dementia and those with a greater extent of pathological lesions (Aβ plaques and neurofibrillary tangles [NFTs]) [62]. However, the exact prevalence of AD in cardiac amyloidosis and vice versa is currently unknown. There is only preliminary evidence in a small single-center cohort that AD patients more frequently show hallmark features of an infiltrative cardiomyopathy (i.e., lower electrocardiographic QRS voltages and voltage/mass ratios) as compared to cognitively normal participants [63]. Moreover, it is unclear whether this may represent a systemic deposition of Aβ [64] or an association between AD and cardiac amyloidosis caused by different precursors.

The brain of patients with AD is characterized by a massive presence of extracellular amyloid plaques and intracellular NFTs. The transmembrane protein APP is cleaved producing Aβ40 and Aβ42, which may form insoluble aggregates [65]. NFTs are made of hyperphosphorylated tau, a microtubule-associate protein that can form insoluble helical filaments; these are thought to cause neuronal death through processes of synaptic disturbance, oxidative stress, and mitochondrial dysfunction. Co-presence of β-amyloid peptide and NFTs in AD [66], and evidence that NTFs formation follows Aβ accumulation [67, 68], led to the amyloid hypothesis, which is still the primary model of AD pathogenesis [69].

## Anti-amyloidogenic Activity of Transthyretin in Alzheimer’s Disease

AD is believed to develop when there is an imbalance between the production and clearance of soluble Aβ (sAβ). The removal of Aβ from the brain requires mostly 3 systems: externalization across the blood-cerebrospinal fluid barrier and blood–brain barrier (BBB), cellular internalization, or enzymatic cleavage [70]. Demonstration that human CSF inhibited Aβ40 aggregation [71] suggested that some molecules can sequester sAβ circulate in the fluid. The main sequestering protein was found to be TTR, followed by ApoE [72]. Once the TTR/Aβ complexes are formed, several pathways can lead to Aβ clearance. First and foremost, TTR acts as a transporter, carrying the peptide outside the CNS through the BBB. TTR may also directly cross the barrier, but only in the brain-to-blood direction, hence promoting a decrease in Aβ levels in the brain. However, in most cases, the passage is mediated by the low-density lipoprotein receptor-related protein 1 (LRP1), the main Aβ efflux receptor [73]. Not only TTR presents the peptide to its receptor on the brain side, but it is also capable of regulating BBB permeability to Aβ by modulating LRP1 externalization in cerebral endothelial cells [74].

Native TTR has a similar affinity for different Aβ configurations: monomers, oligomers, and fibrils [75]. When bound to non-toxic sAβ monomers, it prevents their aggregation and promotes their removal from the CSF [75]. Nonetheless, tetrameric TTR was found also in Aβ oligomers and plaques, possibly following a failed attempt to prevent such structures because of an impaired TTR/Aβ ratio [60].

In vitro experiments demonstrated that even a kinetically stable monomeric variant of TTR (M-TTR) can bind Aβ aggregates, but not Aβ monomers [76]. M-TTR prevents oligomerization and fibrillation by stabilizing the amyloid peptide in non-cytotoxic and non-fibrillar, yet insoluble, deposits: Aggregating the oligomers in larger and more stable compounds is far more efficient than keeping isolated peptides separated [77]. However, since the tetramer is one thousand times more concentrated than the monomer [78], the main AD inhibitor likely remains tetrameric TTR.

The molecular structure of the TTR/Aβ complex has been firstly explored through computational models [72] and then with protein engineering [79]. The binding sites for Aβ have been identified as intra-monomeric superficial domains (near A and G β-strands), as well as the inter-monomeric hydrophobic channel for T4 [80].

Interestingly, TTR has also been characterized as a metalloprotease, whose main substrates are ApoA-I and, in the brain, neuropeptide Y and Aβ [38, 45, 46]. In vitro, TTR is able to cleave Aβ aggregates and decrease their amyloidogenic potential [81]; evidence in vivo is still lacking. Finally, the C99-terminal residue of APP, known as CTFβ, can bind to the TTR hydrophobic pocket instead of T4. In this conformation, γ-secretase is unable to reach APP and operate the cut that would release Aβ in the CSF [82].

**Fig. 3:**
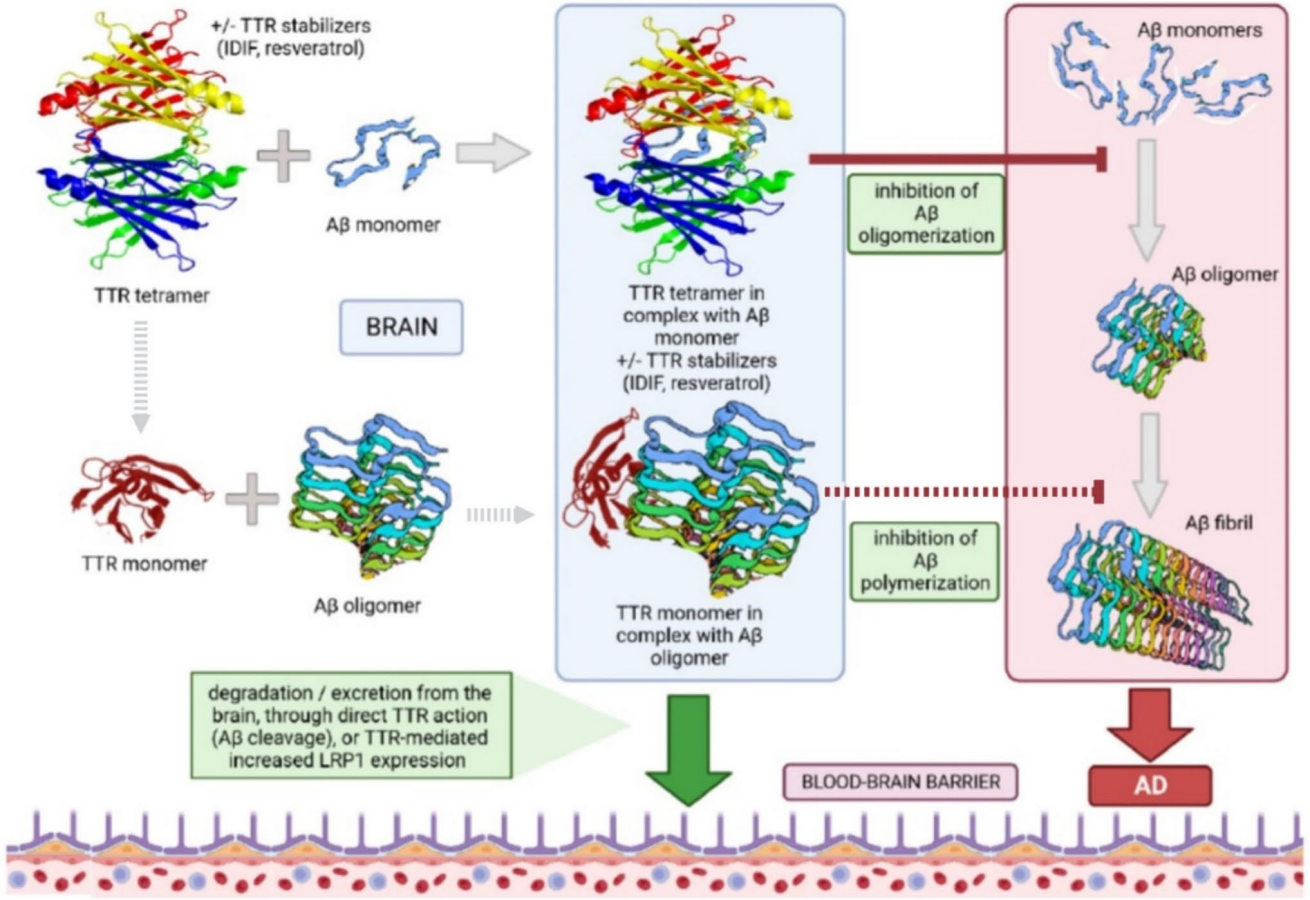
Proposed mechanisms of protection from Aβ fibril depositions by transthyretin (TTR)

All these mechanisms, summarized in Fig. 3, may contribute to Aβ removal from the CNS and explain the neuroprotective roles of TTR in AD. However, further in vitro and in vivo studies are needed to better understand how endogenous factors affect Aβ levels and deposition and to develop new strategies for stabilizing TTR tetramers.

Tetrameric TTR binds Aβ monomers in its hydrophobic channel for T4, inhibiting their oligomerization. TTR monomers are unstable species with very low concentrations in vivo. As demonstrated in vitro through a kinetically stable TTR monomer, the monomeric form can bind Aβ oligomers, inhibiting their polymerization in toxic fibrils. TTR/Aβ complexes are subsequently degraded by TTR through its proteolytic activity, or excreted in the blood flow through LPR1 receptors, whose expression is enhanced by TTR itself.

## Therapeutic Potential of Transthyretin in Alzheimer’s Disease

As explained above, TTR can physiologically bind to Aβ and decrease the concentration of toxic amyloidotic aggregates. M-TTR has proven to be a more powerful anti-oligomerization factor [77]; however, TTR mostly circulates as a tetramer in vivo, and the presence of kinetically stable monomers is quite uncommon. Their affinity to Aβ (and then their anti-AD potential) needs to be specifically evaluated to understand the connection between the two pathologies. In 2004, Schwarzman et al. investigated the affinity to T4 and binding to Aβ of 47 TTR variants. They found an inverse correlation between the amyloidogenic potential of each variant and its ability to sequester Aβ peptides, which suggests that the stability of tetrameric TTR is a fundamental factor in TTR/Aβ interaction. For example, the strongest amyloidogenic variants, P55 and G42, proved totally unable to prevent Aβ polymerization [83]. Therefore, stable TTR tetramers protect not only from ATTR amyloidosis, but from AD as well.

It has been observed that the blood brain barrier (BBB) crossing capability of TTR is only unidirectional, and it cannot traverse BBB from periphery blood to brain [84]. Moreover, the concentration of endogenous TTR in brain seems to be too low to inhibit and transport excessive Aβ during AD progression [85]. For this reason, Wang and colleagues designed a recombinant TTR fused with a cell-penetrating peptide (Pen) to create TP, which significantly enhanced BBB penetration and Aβ inhibition. TP exhibited superior Aβ aggregation inhibition, reduced Aβ-induced toxicity, and extended the lifespan of AD model organisms at low concentrations. Due to its high BBB permeability, TP effectively transports Aβ out of the brain, showing great potential for AD treatment [86].

Different strategies for TTR stabilization have been proposed, starting from TTR stabilizers like tafamidis, dinitrophenol, resveratrol, or iododiflunisal. Tafamidis is a small molecule binding the T4 site and stabilizing the TTR tetramer. Tafamidis treatment has been associated with decreased presence of amyloid plaques and increased Aβ efflux from the brain [87]. Unlike T4, tafamidis does not block Aβ from binding in the central channel, due to its minor dimensions; therefore, Aβ clearance by TTR is preserved. Both dinitrophenol and resveratrol may enhance TTR binding to Aβ, but only resveratrol may increase the proteolytic activity of TTR [88]. In AβPPswe/PS1A246E transgenic mice carrying one copy of the *TTR* gene (AD/TTR ±), iododiflunisal bound TTR in plasma and stabilized the protein, and was able to enter the brain, as revealed by mass spectrometry analysis of CSF. Iododiflunisal administration resulted not only in decreased brain Aβ levels and deposition, but also in improved cognitive function associated with the AD-like neuropathology. Additionally, in AD/TTR ± mice, Aβ levels decreased in plasma, indicating that TTR facilitated Aβ clearance from both the brain and the periphery [89].

## Conclusions

TTR has a tetrameric structure with an intrinsic propensity to disaggregate in monomers; these subsequently re-aggregate in toxic amyloid fibers, accumulating in the heart, kidney, and both peripheral and central nervous system. In the latter, they cause cognitive and functional impairment, similarly to what happens in AD patients. There is growing evidence that Aβ amyloid accumulation in the brain is closely related to a higher risk of AD. Clearance of Aβ is seen as a critical stage to avoid its accumulation into the brain. TTR was described as the major binding site for Aβ in the CNS, and a protective role for it against neurodegenerative diseases and AD was postulated. The tetrameric form of TTR binds Aβ inside its hydrophobic central channel and inhibits the formation of toxic amyloid fibers. Moreover, TTR can increase their degradation directly, through its intrinsic proteolytic action, and indirectly, by restoring the expression of LRP1 and thus facilitating their elimination into the blood flux through the BBB. When a ligand (which can be either T4 or its competitors) is bound to the central hydrophobic channel, the TTR tetramer is less likely to dissociate. Furthermore, if the ligand is a small molecule, it can occupy the T4 binding sites without affecting its capability to bind Aβ, thus enhancing the TTR/Aβ interaction. Interestingly, by binding Aβ peptides, TTR reduces not only their amyloidogenic potential, but also its own. Small T4 competitors have also the ability to cross the BBB and deserve consideration as possible strategies to slow down cognitive decline in AD. Although patients with TTR-FAP who received tafamidis showed a 52% reduction in neurological decline compared to those who received the placebo over an 18-month period [90], no data are currently available on cognitive function of patients on tafamidis over the long term. We may also consider that a massive presence of T4 competitors in the CNS, probably required in cases of severe amyloid deposition, could limit the availability of T4 for neurons.

While the protective effects of tetrameric TTR in AD seem rather well established, significant gaps remain in our understanding that warrant further investigation. Evidence has emerged about the beneficial effects of TTR stabilization in the pathogenesis of AD. However, retrospective clinical studies, reviewed in (14), have been conducted to collect data on a possible correlation between mutated TTR concentration and the prevalence of AD in the sample. The results are controversial, leading the authors to conclude that it is not yet possible to establish a direct correlation between mutated TTR and the onset of AD. Therefore, the cause-effect relationship between TTR instability and AD development remains to be confirmed through mechanistic studies, such as experiments involving mice with TTR mutations or those receiving injections of unstable TTR isoforms. To better establish the protective role of TTR in AD, future research should focus on developing TTR stabilizers, optimizing recombinant TTR proteins for enhanced BBB penetration and Aβ inhibition, and conducting detailed mechanistic studies on TTR/Aβ interactions. Future studies should aim to elucidate molecular mechanisms of action of endogenous factors on Aβ levels and deposition. Preclinical and clinical trials, along with biomarker development and genetic studies, are essential to evaluate the efficacy and safety of these approaches. Additionally, longitudinal studies evaluating patients taking new drugs, such as tafamidis, whose administration has been recently extended in cardiac ATTR, are essential to establish causality and inform clinical practice. Furthermore, exploring the potential therapeutic implications of other molecules described above through randomized controlled trials holds promise for improving patient outcomes. Continued research efforts in these directions will be instrumental in advancing our understanding of the impact of TTR in different sites of the body and translating findings into clinical benefits.

## Data Availability

Not applicable No datasets were generated or analysed during the current study.
